# Basic beliefs of hope: a cross-cultural comparison

**DOI:** 10.3389/fpsyg.2025.1520887

**Published:** 2025-03-05

**Authors:** Andreas M. Krafft

**Affiliations:** Institute of Systemic Management and Public Governance, University of St. Gallen, St. Gallen, Switzerland

**Keywords:** perceived hope, dispositional hope, transdisciplinarity, cross-cultural, basic beliefs

## Abstract

Based on a transdisciplinary concept of hope defined as the belief in the possibility of a wished-for good and the trust in (external) resources that could make this possibility happen, the current paper attempts to evaluate the nature and role of basic beliefs related to a broader perception of hope from people with diverse cultural backgrounds. Two empirical studies from the Hope Barometer research program performed in November 2021 (*N* = 1.721) and November 2023 (*N* = 2.064) aim to compare the levels of generally perceived hope and basic beliefs of the French and Italian populations in Switzerland. Via multivariate hierarchical regression analyses we evaluate the extent to which culturally shaped basic beliefs are distinctively connected to this perception of hope. The results back up the idea that believing in the world’s goodness, fairness, abundance, controllability, and beauty, along with a sense of luck and self-worth, can give people hope that goes beyond just focusing on their own agency and ability to reach their individual goals. Despite similar socio-economic conditions, participants representing the Italian-speaking population display higher levels of perceived hope, dispositional hope, and several basic beliefs about the world and oneself. Furthermore, in the Italian group, primal world beliefs have a stronger connection to perceived hope than in the French speaking group. With regard to psychological theories of hope, these findings imply that it would be misleading to reduce the experience of hope only to individualistic goal-oriented dimensions and to ignore other elements and sources of hope, particularly when hope is related to some broader social domains.

## 1 Introduction

Currently, as crises and conflicts around the world are perceived to be increasingly threatening, a sense of hope becomes more and more indispensable. While the focus of hope is on an individual level in the case of personal crises, societal crises require the understanding of a broader and more general sense of hope (Braithwaite, 2004). Many disciplines, including philosophy, theology, nursing, health sciences, and psychology, have studied the basic attributes and qualities of hope, a fundamental existential phenomenon that people experience in countless contexts and different life situations (Eliott, 2005). Recent research has highlighted the constitutive role of basic beliefs in the experience of hope and demonstrated how people in different cultures can differ in their levels and perceptions of it (Krafft et al., 2023b). Grounded in a transdisciplinary model of hope and based on empirical findings from cross-cultural studies, this paper aims to investigate the fundamental role of basic beliefs as central elements in the experience of hope.

## 2 Theoretical background

### 2.1 A transdisciplinary concept of hope

Most researchers in positive psychology have been conceptualizing and studying hope mainly from an individualistic point of view. The focus of analysis has been the individual disposition to hope, directing attention toward personal goals, a sense of individual agency, and the idea of being able to overcome emerging obstacles and setbacks by one’s own strengths and efforts (Snyder, 2002). Despite its benefits in supporting people to cope with personal challenges, this individualistic conceptualization of hope may be limited when dealing with issues that are only partly or completely out of the hands of the individual. For instance, hoping for a new job for oneself is not the same as hoping for a good job for one’s partner or children, or hoping for the government to create conditions that will generate jobs for people in general. Recent progress in the study of hope psychology has shown that it is necessary to look at hope in a broader, more systemic, and transdisciplinary way (Colla et al., 2022; Krafft et al., 2023c).

Recently, Krafft et al. (2023c) have developed a framework that contains the constitutive elements of hope as a universal phenomenon of human existence, while simultaneously acknowledging the many circumstances in which people in different situations and with diverse cultural backgrounds might hope. Rooted in well-established philosophical theories (see for example Blöser and Stahl, 2017; Callina et al., 2018), hope is being described as comprising three basic elements: (1) a wish or desire for a valued outcome or state of affairs; (2) the belief that its realization is possible, however uncertain and not necessarily probable; and (3) the trust in existing or future resources (such as external support) that enables people to continue hoping especially when confronting obstacles and setbacks. All three elements are necessary conditions for hope but can take a variety of forms. For example, people can wish for personal goals, for the wellbeing of other people close to them (e.g., in case of illness), for their communities or countries (e.g., during a football match), for the natural environment, or even for some transcendent experiences (e.g., the reunion with a loved person in the afterlife). Similarly, the belief in the possibility of its realization can be rooted in basic assumptions and beliefs about the world and oneself: for example, the belief in future opportunities, the belief in common values that foster care and support among people, the belief in the possibilities of modern medicine, the belief in the regenerative power of nature, or the belief in a loving and almighty higher power. Likewise, one can trust in one’s own capabilities, in the concrete assistance of other people, in the abilities of the treating doctor, in the strengths of the team, and so on.

Since the perception of hope can be experienced and fostered in a great variety of ways, hope as a general psychological construct can be understood as a unique and irreducible concept, as philosophically elaborated by Blöser (2019). This means, that, on the one hand, hope must be rooted in the three previously described elements, wish, belief, and trust, but, at the same time, it is an emergent property or phenomenon with different qualities for different people. One aim of the cross-cultural study of hope is to compare mean levels of hope across samples of people from different cultures and to explore the main factors associated with their variance. A central question is, therefore, how to assess hope across several cultures. In this sense, one major challenge is to operationalize the construct of hope to allow cross-cultural comparisons without biasing the research with the researcher’s own values and theories. In order to be able to assess the level of hope as general as possible, it would be necessary to use an instrument to measure the level of hope as perceived by people, avoiding any cultural preconceptions regarding its nature and quality. The Perceived Hope Scale (PHS) was developed for exactly this purpose (Krafft et al., 2019). With the PHS (see section 3.1.2.) it is possible to measure the general level of hope and relate it to different dimensions of hope regarding its possible roots and sources, such as basic beliefs (see for example Krafft et al., 2023b, 2023d, 2023e).

This paper is dedicated to investigating the nature and role of basic beliefs that could foster people’s ability to hope in a general sense, taking special consideration of different cultural backgrounds.

### 2.2 Primal beliefs and basic assumptions

In a general sense, basic beliefs are constitutive elements of worldviews, i.e., assumptions about the nature, quality, and meaningfulness of what and how the world is and why it is as it is (Clifton et al., 2019; Ibrahim, 1984; Janoff-Bulman, 1992; Koltko-Rivera, 2004). Similarly to hope, worldviews, as a set of basic beliefs and assumptions, are especially relevant when one is confronted with the unknown, where uncertainties and inconsistencies arise and people feel an urge to explain the inexplicable (Koltko-Rivera, 2004). According to Janoff-Bulman (1989, 1992), worldviews include basic assumptions or narratives that are related to people’s emotions, thoughts, and behaviors, especially in imagining what could or will happen in the future. In this sense, worldviews serve as theories to anticipate the future and guide the way people interpret new situations.

When people face stressful situations or experiences of powerlessness or despair, basic beliefs become especially relevant in relation to hope (Beck, 1974; Beck et al., 1990). For instance, basic beliefs can define the underlying character of human nature as good or evil and of the world as just or unjust (Lerner, 1980). Therefore, basic beliefs, assumptions about the future, and personal attitudes can shape people’s view of the world and themselves, influencing their perception of hope. In general terms, hope is anchored in the belief that the future can provide new possibilities and current situations can (but not necessarily will) change for the better (Krafft et al., 2023b).

Several authors maintain that the phenomenon of hope must be understood in the context of the culture in which people are embedded (see Averill et al., 1990; Averill and Sundararajan, 2005; Scioli and Biller, 2009; Tennen et al., 2002). Culturally shared worldviews and beliefs about how things are - and how they could and should be - can strongly influence people’s perceptions and volitions (Miller, 1999). From this cultural point of view, individual and social hopes can emerge through shared beliefs, which are constitutive of collective worldviews (Farsani and Abolghasemi, 2008). For instance, higher levels of hope might sometimes be rooted in collective beliefs regarding individual capabilities, control, and efforts (like in many Western societies), the cultural importance of the family and a sense of community (like in several Latin countries), and in some other cases in the common belief in a benevolent higher power (as observed in many African societies) (Krafft et al., 2023a).

For the current studies, we revert to the conceptual and empirical work of Janoff-Bulman (1989, 1992) as well as Clifton and Yaden (2021). For Janoff-Bulman, one of the pioneers in the psychological study of personal beliefs, basic assumptions that are at the core of people’s worldviews can be conceptualized in three primary categories: the perception of the external world, the perception of ourselves, and the perception of the relationship between both. The first category is characterized by the belief in the benevolence of people and the world in general. The fundamental distinction lies in the belief that good prevails over evil or vice versa. When people believe that the world is a good place to live and that people are basically kind, helpful, and caring, they experience lower levels of distress and higher levels of subjective well-being (Janoff-Bulman, 1992; Joseph and Linley, 2005). The second category of basic assumptions is the belief in the meaningfulness of the world. People usually tend to believe that what happens to them and to other people makes sense. Belonging to this category are the basic beliefs of justice, controllability of events, and chance. Frequently, people believe that what happens to them is a consequence of their own attitudes and behaviors. In a just world, people deserve what they experience. This would mean that good people could hope for good outcomes. Furthermore, we have the power to control certain circumstances, while others remain beyond our control. Sometimes, what happens in the world and to oneself can be influenced by our own behavior. The opposite belief is that things happen just by chance. If events occur at random, they will lack any meaning and instill a sense of helplessness, as there is nothing one can do to promote or avoid them. The third fundamental assumption focuses on people’s self-perceptions, encompassing three dimensions. The first dimension is that of self-esteem, which arises when people perceive themselves as good, decent, and therefore worthy individuals. The second dimension refers to the appropriateness and effectiveness of one’s actions in order to be in control of one’s own life. The third belief is that of luck (or misfortune). In many situations, people are not able to control what happens to them, but they nevertheless feel somehow protected from misfortune.

A similar model regarding basic beliefs about the nature and quality of the world was recently developed by Clifton and his colleagues (Clifton et al., 2019; Clifton and Yaden, 2021). Beliefs about the basic character of the world are defined as primal beliefs (also called just primals). Similarly to Janoff-Bulman’s first category, the world can generally be considered as good or bad. People can either believe that the world is a beautiful, fascinating, safe, and exciting place, or to the contrary, that it is dangerous, ugly, and meaningless. Related to the phenomenon of hope, one can either believe that the world is improvable and will get better, or that things are impossible to change and are getting worse. Three overarching secondary primals classify the basic assessment of the world as good or bad: safe versus dangerous, enticing versus dull, and alive versus mechanistic. Individuals can perceive the world as a safe, fair, and benign place, brimming with new and fascinating opportunities, and a place where they can intentionally alter things for the better or not.

In sum, hope can be significantly associated with the belief in the goodness of the world and of people in general, in one’s dignity, self-worth, and capacity to control their own fate. In some cases, people believe in external forces they cannot explain or control, such as luck. Hope can be related to the propensity for a person to believe in a positive future, in a favorable development of life in general, in the social support one receives, and in the appreciation of one’s own capabilities. In our research, we furthermore presumed that people with different cultural backgrounds can hold different worldviews and can consequently experience hope in different ways.

## 3 The current studies

The empirical part of this paper comprises two studies. In both studies, the objective is to assess the basic beliefs of two culturally diverse groups of people in relation to their perceived level of hope. Switzerland is a country with several geographically and culturally demarcated regions. Approximately 67% of the population resides in the German (central and east) region, 23% in the French (west) region, and 8% in the Italian (south) region, with the Romansch minority constituting the fourth cultural group. In several studies, the results showed that the hope levels of the German- and Italian-speaking population are significantly higher than those of the French-speaking people (Krafft, 2019, 2020, 2021). Other publications (Krafft et al., 2019; Krafft and Walker, 2018) have already extensively reported the results of the German-speaking population, thus the present studies will focus on original data from the French and Italian populations, which share similar socio-economic circumstances but have different cultural backgrounds. The research question is whether culturally shaped basic beliefs of people in these two Swiss regions could have a distinct connection with the levels of hope of their population. The first study aims to evaluate the role of primal beliefs, as conceptualized and operationalized by Clifton and Yaden (2021), in relation to hope. In the second study, we assess how basic assumptions about the world and oneself, as defined and operationalized by Janoff-Bulman (1989, 1992), relate to hope. Furthermore, we take the opportunity to assess the psychometric properties of the French and the Italian versions of the Perceived Hope Scale (PHS), which have not been validated and reported yet in any scientific publication.

### 3.1 Study 1

#### 3.1.1 Objectives, procedure and participants

The purpose of the first study is to investigate the role of primal world beliefs as operationalized by Clifton and Yaden (2021) with regard to the perceived levels of hope in two samples of French and Italian-speaking people in Switzerland. In order to cover not only the general beliefs about the world but also the personal disposition to hope, we included the Adult Dispositional Hope Scale (ADHS) of Snyder et al. (1991) into the survey. By doing so, it is possible to analyze whether basic beliefs about the world could have a connection with the more general perception of hope beyond individual traits.

Data was collected through the annual online survey of the Hope Barometer in November 2021. Participants were recruited through newspapers via online advertisements, social media, and e-mails. No incentives were offered. The inclusion criterion was a minimum age of 18. The samples are not strictly representative of the demographic distribution of the Swiss population in the French and Italian regions but include a high variety of people of different ages, education levels, family status, occupation, and professional level. 1,146 participants (66.6%) answered the French questionnaire and 575 (33.3%) answered the Italian questionnaire. Table 1 presents the demographic structure of the two samples. Due to a lack of completely equal demographic distribution, all demographic attributes will be used as control variables in the analytical statistics.

**Table 1:** Demographic structure of the samples.

	French		Italian		Total	
	N/n	%	N/n	%	N/n	%
Total	1,146	100%	575	100%	1721	100%
Age	M = 50.05	SD = 13.78	M = 47.26	SD = 14.29	M = 49.12	SD = 14.01
Gender						
Male	514	44.9%	270	47.0%	784	45.6%
Female	626	54.6%	303	52.7%	929	54.0%
Other	6	0.5%	2	0.3%	8	0.5%
Education						
Did not finish school	10	0.9%	4	0.7%	14	0.8%
Elementary school	64	5.6%	8	1.4%	72	4.2%
Secondary school	76	6.6%	48	8.3%	124	7.2%
Vocational training	612	53.4%	344	59.8%	956	55.5%
Bachelor degree	142	12.4%	63	11.0%	205	11.9%
Master degree	208	18.2%	91	15.8%	299	17.4%
PhD	34	3.0%	17	3.0%	51	3.0%
Family status						
Still living with parents	38	3.3%	35	6.1%	73	4.2%
Single	157	13.7%	85	14.8%	242	14.1%
In a partnership living separately	67	5.8%	53	9.2%	120	7.0%
Living together in a partnership	284	24.8%	102	17.7%	386	22.4%
Married	415	36.2%	228	39.7%	643	37.4%
Divorced / separated	155	13.5%	58	10.1%	213	12.4%
Widowed	30	2.6%	14	2.4%	44	2.6%
Children						
Childless	393	34.3%	271	47.1%	664	38.6%
With children	753	65.7%	304	52.9%	1,057	61.4%
Main occupation						
In training	41	3.6%	20	3.5%	61	3.5%
Family, housework, raising children	49	4.3%	39	6.8%	88	5.1%
Part-time employment	252	22.0%	102	17.7%	354	20.6%
Full-time employment	504	44.0%	302	52.5%	806	46.8%
Unemployed	55	4.8%	29	5.0%	84	4.9%
Retired	245	21.4%	83	14.4%	328	19.1%
Professional status						
No position in a professional organization	204	17.8%	84	14.6%	288	16.7%
In training	55	4.8%	35	6.1%	90	5.2%
Employee	473	41.3%	256	44.5%	729	42.4%
Middle management	190	16.6%	93	16.2%	283	16.4%
Senior management	112	9.8%	34	5.9%	146	8.5%
Owner / Entrepreneur / Self-employed	112	9.8%	73	12.7%	185	10.7%

#### 3.1.2 Measures

The measures included in the study are the Perceived Hope Scale (PHS), the Adult Dispositional Hope Scale (ADHS), and the Primals Inventory short form (PI-18).

##### 3.1.2.1 Perceived hope scale (PHS)

The PHS is a self-rating instrument that was developed to measure the level of hope as perceived by the participants, avoiding any cultural bias regarding the nature and quality of hope (Krafft et al., 2019). The PHS does not measure future expectations of individual goal attainment nor different dimensions of hope regarding its roots and sources (such as agency or self-confidence). The PHS is a unidimensional measure including six positively worded items to be rated on a 6-point Likert scale ranging from 0 (strongly disagree) to 5 (strongly agree). The six items cover the general level of hope (e.g., “I feel hopeful”), the belief in the fulfillment of one’s hopes, whether hope outweighs anxiety and improves the quality of one’s life, and if one can remain hopeful even in difficult times. In the validation study of the original German scale the six items achieved high internal consistency with Cronbach *α* values between 0.87 and 0.89.

##### 3.1.2.2 Adult dispositional hope scale (ADHS)

The ADHS is an instrument to measure the level of hope represented by the self-assessment of one’s own determination (*agency* or willpower) and ability (*pathways* or waypower) to achieve personal goals (Snyder et al., 1991). The concept of dispositional hope is particularly adept at illustrating and individual’s level of self-efficacy and self-confidence. The ADHS consists of 12 positively worded items, 4 items targeting the dimension of agency, 4 items representing the dimension pathways, and 4 distractors (excluded from the analysis). In this study, we used a 6-point Likert-type scale from 0 (strongly disagree) to 5 (strongly agree). The internal consistency of the total English scale as reported in the validation study ranged between 0.74 and 0.84. In the current study the Cronbach Alpha value was *α* = 0.93 in both samples.

##### 3.1.2.3 Primals inventory short form (PI-18)

Derived from the original Primals Inventory (PI-99), Clifton and Yaden (2021) developed a Primals Inventory short form (PI-18) as a parsimonious instrument to measure the higher-order world beliefs represented by the dimensions *Safe*, *Enticing*, and *Alive*. Together, the combined score illustrates how *Good* or *Bad* the participants consider the world to be. Six items cover the belief in a safe or dangerous world, with three describing the world’s goodness and safety, and three expressing its danger and potential worsening in the future. Seven items describe how enticing or dull the world is, with four portraying its beauty, fascination, and abundance, and three illustrating its boredom and monotony. Whether the world is perceived as alive or mechanistic is assessed with five items expressing how purposeful it is (with one negatively worded item). The assessment was done using a 6-point Likert scale ranging between 0 (strongly disagree) and 5 (strongly agree). Reliability scores in the English validation study achieved values of *α* = 0.88 for *Good*, α = 0.83 for *Safe*, α = 0.83 for *Enticing*, and *α* = 0.85 for *Alive*. In the current study the internal consistency scores in the French/Italian samples were as follows: the internal consistency scores for *Good* were *α* = 0.84/0.82, for *Safe α* = 0.84/0.82, for *Enticing α* = 0.80/0.78, and for *Alive α* = 0.52/0.56.

#### 3.1.3 Data analysis

The statistical analyses were performed with IBM SPSS and AMOS version 29.0 and completed in four steps:

Step 1: The psychometric properties and group invariance of the PHS, which is the main tool used to measure the general level of hope, had to be checked before the mean levels of hope between the two samples could be compared. We conducted the assessments using confirmatory factor analyses with maximum likelihood estimations, the Cronbach alpha indicator for internal consistency, and multigroup factor analyses with maximum likelihood estimations to verify group invariance.

Step 2: In the second step, mean values and standard deviations were calculated for all variables. The mean values of both samples were then compared via analysis of variance (ANOVA).

Step 3: Through Pearson partial bivariate correlations, the relationships between the hope variables and primal beliefs were calculated (after controlling for demographic variables) and then compared via Fischer’s *z*-tests.

Step 4: Finally, multivariate hierarchical regressions were calculated to predict perceived hope in both samples separately. Following the research question, the purpose of the analysis is to identify whether basic beliefs about the world constitute an additional predictor beyond dispositional hope, resulting in higher *R* ^2^ scores of explained variance in perceived hope, and whether the effects could vary between the two samples. Comparing the effects of predictive power and explained variance between both groups can offer a possible explanation for the different score levels of perceived hope among the French- and Italian-speaking populations.

#### 3.1.4 Results

##### 3.1.4.1 Step 1: psychometric properties and group invariance of the PHS

The first step consisted of assessing the one-dimensionality of the PHS in the French and Italian samples separately via CFA. In both cases, all six items revealed significant and high loading estimates on the latent variable, between 0.69 and 0.90 in the French sample and between 0.70 and 0.92 in the Italian sample. The models in both samples displayed a good fit to the data [as recommended by Hu and Bentler (1999)], with Comparative Fix Indices (RFI) and Tucker-Lewis Indices (TLI) above 0.95 and root mean squares of approximation (RMSEA) and standardized root mean square residuals (SRMR) equal to or below 0.08 (see Table 2). Furthermore, the six items of the PHS demonstrated high internal consistency with Cronbach Alpha values of *α* = 0.90 in the French sample and *α* = 0.92 in the Italian sample.

**Table 2:** Model fit and group invariance of the French and the Italian versions of the PHS.

	*X* ^2^	df	CFI	TLI	RMSEA	SRMR
CFA French PHS	68.95	9	0.973	0.977	0.076	0.0199
CFA Italian PHS	41.78	9	0.972	0.978	0.080	0.0184
Group invariance						
Configurational invariance (equal form)	119.72	23	0.976	0.981	0.049	0.020
Metric invariance (equal loadings)	220.43	29	0.966	0.971	0.062	0.020
Scalar invariance (equal intercepts)	222.7	30	0.967	0.971	0.061	0.022
Strict invariance (equal residuals)	249.4	36	0.969	0.974	0.059	0.024

CFA, Confirmatory Factor Analysis; CFI, Comparative Fix Index; TLI, Tucker-Lewis Index; RMSEA, Root Mean Square of Approximation; SRMR, Standardized Root Mean Square Residual; PHS, Perceived Hope Scale.

The multi-group CFA results are shown in Table 2, along with the fit indices of four models used to test strict, metric, and scalar invariance. We used the equal form as a baseline model, which provided a good fit to the data, suggesting reasonable support for configurational invariance across both groups. Likewise, all indices comparing the further models with the baseline model were under the threshold values recommended by the literature (Chen, 2007, CFI and TLI > -0.01, RMSEA and SRMR <0.015). This indicates that the PHS demonstrated a strong invariance among the French- and Italian-speaking populations, allowing for a comparison of the PHS scores between the two samples. This makes them suitable for further examination in relation to other constructs.

##### 3.1.4.2 Step 2: descriptive statistics and mean scores comparisons

Table 3 displays the mean values and standard deviations of the six variables included in the analysis. The first finding is that people in both samples revealed moderate levels of perceived hope and slightly higher levels of dispositional hope. Participants in the Italian sample reported significantly higher levels of perceived and dispositional hope than people in the French sample. Furthermore, mean values of the primal beliefs were also moderate, with similar scores between samples in the dimensions *Good* and *Safe*, and slightly higher scores in the Italian sample in the dimensions *Enticing* and *Alive*.

**Table 3:** Descriptive statistics and mean scores comparisons.

	French		Italian		Total		ANOVA	
	Mean	SD	Mean	SD	Mean	SD	F	*p*
Perceived hope	2.914	1.183	3.132	1.251	2.986	1.210	12.521	<0.001
Dispositional hope	3.250	1.030	3.700	0.955	3.400	1.028	76.910	<0.001
Primal-Good	2.799	0.755	2.868	0.734	2.822	0.748	3.265	0.071
Primal-Safe	2.464	1.007	2.419	1.003	2.449	1.005	0.750	0.386
Primal-Enticing	3.325	0.872	3.409	0.815	3.353	0.854	3.762	0.053
Primal-Alive	2.464	0.941	2.648	0.965	2.526	0.953	14.324	<0.001

This means that the Italian participants tend to perceive the future more hopefully, both in general terms as well as with regard to their personal goals, and that they also envision the world as being somewhat more interesting, abundant, improvable, and at the same time less mechanistic or distant.

##### 3.1.4.3 Step 3: partial bivariate correlations

The partial bivariate correlation scores between primal beliefs and perceived and dispositional hope, along with Fischer’s correlation comparison effects (z), are represented in Table 4. Basically, all correlation values are significant and of moderate to higher magnitude. In both samples, the correlation coefficients of the primal beliefs with the PHS are higher than those with the ADHS, which means that the primal beliefs are more strongly related to the broader perception of general hope than with the willpower and personal capacity directed to achieving one’s own goals. In the French sample, the correlation score between the PHS and the ADHS is significantly higher than in the Italian sample. Furthermore, while the Italian sample shows a stronger relationship between the primal dimension *Safe* and the PHS (statistically not significant), the French sample exhibits a larger magnitude relationship between the primal dimension *Enticing* and the PHS. In terms of dispositional hope, the French sample displays higher correlation scores with the general primal *Good* and, more specifically, with the primal dimension *Enticing* than the Italian sample.

**Table 4:** Partial bivariate correlations and Fischer’s correlation comparisons.

	Perceived Hope Scale (PHS)				Adult Dispositional Hope Scale (ADHS)			
	French	Italian	Correlation comparisons		French	Italian	Correlation comparisons	
	Perceived hope		z	*p*	Dispositional hope		z	*p*
ADHS	0.663^**^	0.566^**^	3.05	0.002	-	-	-	-
Primal-Good	0.546^**^	0.545^**^	0.03	0.976	0.491^**^	0.398^**^	2.27	0.023
Primal-Safe	0.389^**^	0.449^**^	−1.42	0.155	0.331^**^	0.286^**^	0.97	0.332
Primal-Enticing	0.499^**^	0.401^**^	2.40	0.016	0.467^**^	0.332^**^	3.15	0.002
Primal-Alive	0.423^**^	0.449^**^	−0.63	0.529	0.381^**^	0.335^**^	1.03	0.303

** Correlation is significant at 0.01 level; control variables: Gender, age, family status, children, education, main activity and professional status.

In summary, the French-speaking population associates hope more strongly with an individual’s agency and ability to achieve personal goals, along with their perception of the world as an interesting, improvable, and abundant place. On the other hand, for the Italian-speaking population, the belief in the world as safe, cooperative, and stable has a stronger connection to their general perception of hope than for their French-speaking counterparts. Furthermore, the individual capacity to achieve one’s own goals is more strongly related to the belief in an enticing (interesting, abundant, meaningful) world.

##### 3.1.4.4 Step 4: multivariate hierarchical regression analyses

Before presenting the results of the regression analyses, it is worth reporting that all variables were tested for collinearity. All variance inflation factors (VIF) were far away from indicating any collinearity (VIF between 1.26 and 1.65 and tolerance between 0.61 and 0.79).

In the first multivariate hierarchical regression analyses, the demographic variables were entered in the first equation, and subsequently, the three primal dimensions were added in step 2. The results in Table 5 demonstrate that in both samples all models were significant and that the three primal factors explain 30.1% of the variance of the PHS in the French sample and 27.8% in the Italian sample.

**Table 5:** Regression models with demographics and primals.

Dependent variable: Perceived hope (PHS)	French			Italian		
	Adj. *R*^2^	Adj. *R*^2^ Δ	Sig.	Adj. *R*^2^	Adj. *R*^2^ Δ	Sig.
Model 1 - Demographics	0.052	0.052	<0.001	0.052	0.052	<0.001
Model 2 - Demographics + Primals	0.353	0.301	<0.001	0.330	0.278	<0.001

In regard to the detailed indicators in Model 2, the primal *Enticing* was the strongest predictor of the PHS in the French sample (followed by *Alive* and *Safe*), whereas *Alive and Safe, followed by Enticing, were the strongest predictors* in the Italian sample (Table 6).

**Table 6:** Multivariate hierarchical regression analyses with primal dimensions.

Dependent variable: Perceived hope (PHS)	French			Italian		
Model 2 (*p* < 0.001)	Std. *ß*	*t*	Sig.	Std. *ß*	*t*	Sig.
Step 1: Demographics						
Gender	−0.011	−0.435	0.664	0.020	0.537	0.592
Age	0.179	5.779	<0.001	0.113	2.546	0.011
Family status	0.017	0.611	0.542	0.033	0.745	0.456
Children	0.059	2.084	0.037	0.041	0.902	0.367
Education	−0.056	−2.239	0.025	0.013	0.360	0.719
Main activity	−0.026	−0.898	0.369	0.019	0.484	0.628
Professional status	0.045	1.791	0.074	−0.003	−0.084	0.933
Step 2: Primals						
Primal-Safe	0.080	2.599	0.009	0.243	5.549	<0.001
Primal-Enticing	0.355	11.651	<0.001	0.178	4.333	<0.001
Primal-Alive	0.258	9.468	<0.001	0.259	6.497	<0.001

In the second round of regression analyses, the demographic variables were entered in step 1, followed by the ADHS in step 2, and the global primal *Good* in step 3. Results in Table 7 express that all models were significant in both samples. Dispositional hope (ADHS) explains 41% of the perceived hope’s (PHS) variance in the French group and 30.1% in the Italian group. In addition, the general primal belief *Good* explains 6.3% in the French and 11.1% of the PHS’s variance beyond the ADHS.

**Table 7:** Multivariate hierarchical regression models with demographics, ADHS, and primal.

Dependent variable: Perceived hope (PHS)	French			Italian		
	Adj. *R*^2^	Adj. *R*^2^ Δ	Sig.	Adj. *R*^2^	Adj. *R*^2^ Δ	Sig.
Model 1 - Demographics	0.052	0.052	<0.001	0.052	0.052	<0.001
Model 2 - Demographics + ADHS	0.462	0.410	<0.001	0.353	0.301	<0.001
Model 3 - Demographics + ADHS + Primal-Good	0.525	0.063	<0.001	0.464	0.111	<0.001

Table 8 presents the detailed effect sizes of the single variables predicting perceived hope in Model 3. The effect of the individual goal oriented dispositional hope on the broader concept of perceived hope is stronger in the French sample than in the Italian one. On the other hand, the effect of the primary belief *Good* on perceived hope is more accentuated in the Italian sample than in the French sample.

**Table 8:** Multivariate hierarchical regression analyses with dispositional hope and the overall primal “Good.”

Dependent variable: Perceived hope (PHS)	French			Italian		
Model 3 (*p* < 0.001)	Std. *ß*	*t*	Sig.	Std. *ß*	*t*	Sig.
Step 1: Demographics						
Gender	0.031	1.444	0.149	0.042	1.265	0.206
Age	0.142	5.354	<0.001	0.067	1.694	0.091
Family status	−0.004	−0.181	0.856	−0.003	−0.083	0.934
Children	0.035	1.466	0.143	0.038	0.941	0.347
Education	−0.084	−3.966	<0.001	−0.027	−0.847	0.397
Main activity	−0.047	−1.89	0.059	0.040	1.125	0.261
Professional status	−0.015	−0.689	0.491	−0.027	−0.847	0.397
Step 2: Dispositional hope	0.515	21.255	<0.001	0.418	12.014	<0.001
Step 3: Primal-Good	0.296	12.285	<0.001	0.373	10.853	<0.001

#### 3.1.5 Discussion study 1

These results indicate that the broader perception of hope is significantly related to both self-assessed willpower and the ability to achieve one’s own personal goals, as well as to the belief in the goodness of the world. Interestingly, in their effects on perceived hope, in the French group the effect size of the personal disposition is higher than in the Italian group, whereas in the Italian group the effect size of the primal belief *Good* is considerably stronger than in the French sample. This could be a possible explanation for why the Italian population reported having higher levels of perceived hope.

### 3.2 Study 2

#### 3.2.1 Objectives, procedure, and participants

The purpose of the second study is to examine the role of basic beliefs about the world and oneself as conceptualized and operationalized by Janoff-Bulman (1989, 1992) with regard to the perceived levels of hope in two other samples of French- and Italian-speaking people in Switzerland.

Data was collected through the annual online survey of the Hope Barometer in November 2023. Participants were recruited through newspapers via online advertisement, social media, and e-mails. No incentives were offered. The inclusion criterion was a minimum age of 18. The samples are not strictly representative of the demographic distribution of the Swiss population in the French and Italian regions, but include a high variety of people of different ages, education levels, family status, occupation and professional level. 1,458 participants (70.6%) answered the French questionnaire and 606 (29.4%) answered the Italian questionnaire. Table 9 presents the demographic structure of the two samples. Due to a lack of completely equal demographic distribution, all demographic attributes will be used as control variables in the analytical statistics.

**Table 9:** Demographic structure of the samples.

	French		Italian		Total	
	N/n	%	N/n	%	N/n	%
Total	1,458	100%	606	100%	2064	100%
Age	*M* = 52.29	SD = 38.83	*M* = 52.10	SD = 38.53	*M* = 52.23	SD = 38.73
Gender						
Male	725	49.7%	331	54.6%	1,056	51.2%
Female	723	49.6%	272	44.9%	995	48.2%
Other	10	0.7%	3	0.5%	13	0.6%
Education						
Did not finish school	14	1.0%	11	1.8%	25	1.2%
Elementary school	71	4.9%	6	1.0%	77	3.7%
Secondary school	55	3.8%	50	8.3%	105	5.1%
Vocational training	624	42.8%	357	58.9%	981	47.5%
Bachelor degree	283	19.4%	78	12.9%	361	17.5%
Master degree	411	28.2%	104	17.2%	515	25.0%
Family status						
Still living with parents	29	2.0%	23	3.8%	52	2.5%
Single	224	15.4%	89	14.7%	313	15.2%
In a partnership living separately	108	7.4%	52	8.6%	160	7.8%
Living together in a partnership	319	21.9%	114	18.8%	433	21.0%
Married	555	38.1%	254	41.9%	809	39.2%
Divorced / separated	192	13.2%	62	10.2%	254	12.3%
Widowed	31	2.1%	12	2.0%	43	2.1%
Children						
Childless	522	35.8%	275	45.4%	797	38.6%
With children	936	64.2%	331	54.6%	1,267	61.4%
Main occupation						
In training	30	2.1%	9	1.5%	39	1.9%
Family, housework, raising children	61	4.2%	39	6.4%	100	4.8%
Part-time employment	312	21.4%	90	14.9%	402	19.5%
Full-time employment	678	46.5%	319	52.6%	997	48.3%
Unemployed	63	4.3%	33	5.4%	96	4.7%
Retired	314	21.5%	116	19.1%	430	20.8%
Professional status						
No position in a professional organization	220	15.1%	91	15.0%	311	15.1%
In training	45	3.1%	19	3.1%	64	3.1%
Employee	634	43.5%	258	42.6%	892	43.2%
Middle management	297	20.4%	119	19.6%	416	20.2%
Senior management	150	10.3%	84	13.9%	234	11.3%
Owner / Entrepreneur / Self-employed	112	7.7%	35	5.8%	147	7.1%

#### 3.2.2 Measures

The measures included in this study are the PHS as described in Study 1 (see section 3.1.2) and the World Assumptions Scale (WAS).

##### 3.2.2.1 World assumptions scale (WAS)

The WAS, developed by Janoff-Bulman (1989), consists of 32 items describing basic assumptions about the world and oneself. Such assumptions correspond to three basic categories and eight dimensions: (1) assumptions about the goodness of the world and of people (which are merged into one indicator as recommended by Elklit et al., 2007); (2) assumptions about the meaningfulness of what is happening in this world, including the dimensions justice, controllability of the world, and randomness; and (3) assumptions about oneself, comprising self-worth, self-control, and luck. Participants were asked to respond to the items on a 6-point Likert scale from 0 (strongly disagree) to 5 (strongly agree). In the current study, the internal consistency scores of the seven dimensions in the French and Italian samples were just acceptable to good: *Benevolence α* = 0.85 and 0.86, *Justice* α = 0.67 and 0.47, *Controllability of the World* α = 0.72 and 0.76, *Randomness* α = 0.69 and 0.57, *Self-worth* α = 0.75 and 0.76, *Self-control* α = 0.67 and 0.61, and *Luck* α = 0.88 in both samples.

#### 3.2.3 Data analysis

As in Study 1, the statistical analyses were performed with IBM SPSS and AMOS version 29.0 and completed in four steps:

Step 1: First, the psychometric properties and group invariance of the PHS were evaluated by means of confirmatory factor analyses with maximum likelihood estimations to test the scale’s dimensionality, the Cronbach’s Alpha Indicator to check its internal consistency, and multigroup factor analyses also with maximum likelihood estimations to check invariance across the French and Italian samples.

Step 2: In the second step, mean values and standard deviations were calculated for all indicators. The mean values of both samples were then compared via analysis of variance (ANOVA).

Step 3: Through Pearson partial bivariate correlations, the relationships between Perceived Hope and the seven dimensions of the WAS were calculated (after controlling for demographic variables) and compared between groups via Fischer’s z-tests.

Step 4: Finally, multivariate hierarchical regressions were calculated to predict perceived hope in both samples separately. The purpose of the analysis is to identify whether, and in how far, the assumptions about the world and oneself predict the levels of perceived hope and to what extent the effects vary between the two samples.

#### 3.2.4 Results

##### 3.2.4.1 Step 1: psychometric properties and group invariance of the PHS

In order to replicate the evaluation done in Study 1, the first step consisted of assessing the one-dimensionality of the PHS in the French and Italian samples separately via CFA. Each of the six items showed moderate to high loading estimates on the latent variable, ranging from 0.72 to 0.90 in the French sample and from 0.67 to 0.90 in the Italian sample (all statistically significant). The models in both groups displayed a good fit to the data [as recommended by Hu and Bentler (1999)], with CFI and TLI above 0.95 and SRMR below 0.08 (see Table 10). The RMSEA slightly exceeded the recommended threshold value of 0.08. However, after correlating the residuals of items 4 (hope and quality of life) and 5 (hope for one’s life) the RMSEA improved to 0.066 in the French and 0.064 in the Italian sample. Furthermore, the six items of the PHS demonstrated high internal consistency with Cronbach Alpha values of *α* = 0.91 in both samples.

**Table 10:** Model fit and group invariance of the French and the Italian versions of the PHS.

	*X* ^2^	df	CFI	TLI	RMSEA	SRMR
CFA French PHS	102.44	9	0.984	0.973	0.084	0.022
CFA Italian PHS	56.766	9	0.980	0.967	0.094	0.025
Group invariance						
Configurational invariance (equal form)	168.9	23	0.982	0.977	0.055	0.022
Metric invariance (equal loadings)	218.57	29	0.977	0.976	0.056	0.022
Scalar invariance (equal intercepts)	221.24	30	0.976	0.976	0.056	0.023
Strict invariance (equal residuals)	262.91	36	0.972	0.977	0.055	0.025

CFA, Confirmatory Factor Analysis; CFI, Comparative Fix Index; TLI, Tucker-Lewis Index; RMSEA, Root Mean Square of Approximation; SRMR, Standardized Root Mean Square Residual; PHS, Perceived Hope Scale.

The multi-group CFA results are shown in Table 10, along with the fit indices of four models used to test the different kinds of invariances. The equal form used as baseline model provided a good fit to the data, suggesting reasonable support for configurational invariance across both groups. Likewise, all indices comparing the further models with the baseline model were under the threshold values recommended by the literature (Chen, 2007, CFI and TLI > -0.01, RMSEA and SRMR <0.015). This means, as already shown in Study 1, that the PHS reveals strong invariance between the French and the Italian groups and that it is possible to compare the PHS scores between the two samples, making them also suitable for further examination in relation to the other variables.

##### 3.2.4.2 Step 2: descriptive statistics and mean scores comparisons

Table 11 displays the mean values and standard deviations of the variables included in the analysis. Similarly to the results in Study 1, participants in both samples revealed moderate levels of perceived hope, with significantly higher levels in the Italian sample compared to the French sample. Furthermore, mean values of basic beliefs were also moderate, with higher scores in *Self-worth* and *Self-control* and lower scores in *Justice*, *Benevolence,* and *Controllability of the World*. Comparing both samples, the levels of *Benevolence* and *Self-worth* were similar, but the Italian participants scored higher in *Justice*, *Controllability of the World*, *Self-Control* (to a low extent) and particularly in the self-assessment of the degree of *Luck* in one’s life.

**Table 11:** Descriptive statistics and mean scores comparisons.

	French		Italian		Total		ANOVA	
	Mean	SD	Mean	SD	Mean	SD	F	*p*
Perceived hope	2.743	1.179	2.960	1.239	2.807	1.201	13.97	<0.001
Benevolence	2.179	0.911	2.194	0.936	2.184	0.918	0.10	0.750
Justice	1.448	0.930	1.837	0.847	1.562	0.924	78.56	<0.001
Controllability of the world	2.134	0.922	2.323	0.981	2.189	0.944	17.43	<0.001
Randomness	2.540	1.030	2.639	0.888	2.569	0.991	4.31	0.038
Self-worth	3.846	0.907	3.797	0.932	3.831	0.915	1.24	0.266
Self-control	2.931	0.801	3.079	0.753	2.975	0.789	15.14	<0.001
Luck	2.146	1.186	2.955	1.131	2.384	1.227	204.09	<0.001

In sum, as in Study 1, the Italian participants display higher levels of perceived hope. Furthermore, they consider the world to be more just and controllable, and they see themselves as having been luckier in life.

##### 3.2.4.3 Step 3: partial bivariate correlations

The results in Table 12 display the partial bivariate correlation coefficients between the seven dimensions of basic beliefs and perceived hope as well as Fischer’s correlation comparison effects (z). Perceived hope correlates positively with all basic beliefs with the exception of randomness. The more people believe in a benevolent, just, and controllable world, as well as in themselves (in terms of self-worth and luck), and the less they believe in pure chance and unpredictability, the more hopeful they are. Comparing the correlation scores of both samples, nearly all effects were similar. Just the score between self-control and perceived hope is significantly higher in the French sample.

**Table 12:** Partial bivariate correlations and Fischer’s correlation comparisons.

	Perceived hope		Correlation comparisons	
	French *r CI 95% (Lo/Up)*	Italian *r CI 95% (Lo/Up)*	*z*	*p*
Benevolence	0.523^**^ (0.482/0.562)	0.461^**^ (0.382/0.532)	1.69	0.091
Justice	0.378^**^ (0.334/0.424)	0.317^**^ (0.246/0.386)	1.43	0.153
Controllability of the world	0.295^**^ (0.244/0.346)	0.317^**^ (0.233/0.397)	−0.5	0.617
Randomness	−0.102^**^ (−0.156/−0.048)	−0.161^**^ (−0.254/−0.073)	1.24	0.215
Self-worth	0.407^**^ (0.359/0.455)	0.390^**^ (0.308/0.462)	0.42	0.674
Self-control	0.323^**^ (0.271/0.373)	0.226^**^ (0.145/0.315)	2.17	0.030
Luck	0.447^**^ (0.403/0.489)	0.454^**^ (0.378/0.531)	−0.18	0.857

** Correlation is significant at 0.01 level; control variables: Gender, age, family status, children, education, main activity, and professional status.

##### 3.2.4.4 Step 4: multivariate hierarchical regression analyses

The statistical check performed in the multivariate regression analyses shows no cases of collinearity between the indicators (VIF between 1.11 and 1.70 and tolerance between 0.59 and 0.90).

All demographic variables were entered in the first step and the seven basic beliefs of the WAS were added in the equation in the second step. The results in Table 13 depict that all models were significant in both samples and that the basic beliefs explain 44.3% of the variance of the PHS in the French sample and 37.4% in the Italian sample.

**Table 13:** Regression models with demographics and basic beliefs.

Dependent variable: Perceived hope (PHS)	French			Italian		
	Adj. *R*^2^	Adj. *R*^2^ Δ	Sig.	Adj. *R*^2^	Adj. *R*^2^ Δ	Sig.
Model 1 - Demographics	0.031	0.031	<0.001	0.059	0.059	<0.001
Model 2 - Demographics + WAS	0.474	0.443	<0.001	0.433	0.374	<0.001

The detailed indicators in Model 2 reveal that three salient predictors of perceived hope are the belief in the *Benevolence* of the world, *Self-worth*, and the experience of having *Luck* in life (Table 14). Whereas the effects of *Self-worth* and *Benevolence* are slightly stronger in the French group, the effect of *Luck* is more pronounced in the Italian sample. Not or barely significant are the beliefs in the *Controllability of the world* and *Self-control*.

**Table 14:** Multivariate hierarchical regression analyses with basic beliefs.

Dependent variable: Perceived hope (PHS)	French			Italian		
Model 2 (*p* < 0.001)	Std. *ß*	*t*	Sig.	Std. *ß*	*t*	Sig.
Step 1: Demographics						
Gender	−0.035	−1.742	0.082	−0.012	−0.37	0.712
Age	0.031	1.577	0.115	0.055	1.7	0.090
Education	−0.052	−2.614	0.009	0.013	0.416	0.677
Family Status	−0.005	−0.236	0.814	0	−0.001	0.999
Children	0.013	0.589	0.556	0.021	0.566	0.571
Main activity	−0.06	−2.96	0.003	−0.036	−1.112	0.267
Professional status	0.028	1.384	0.166	0.073	2.273	0.023
Step 2: Basic beliefs						
Benevolence	0.324	14.525	<0.001	0.255	7.268	<0.001
Justice	0.117	4.885	<0.001	0.120	3.24	0.001
Controllability of the world	0.005	0.188	0.851	0.063	1.46	0.145
Randomness	−0.076	−3.808	<0.001	−0.071	−2.214	0.027
Self-worth	0.254	12.147	<0.001	0.238	6.882	<0.001
Self-control	0.112	4.915	<0.001	0.008	0.199	0.842
Luck	0.224	10.036	<0.001	0.261	7.244	<0.001

#### 3.2.5 Discussion study 2

This study’s main findings indicate that the perception of hope primarily stems from a fundamental belief in the benevolence of people and the world, like the findings of Study 1, as well as a self-assessment of self-worth and luck, rather than a sense of control over the world and one’s own life. Whereas the beliefs of *Benevolence* and *Self-worth* are of similar magnitude in the French and the Italian population, people in the Italian group display significantly higher levels of *Luck*. This could potentially explain the higher levels of hope among the Swiss-Italian population.

## 4 General discussion

The purpose of this paper was to investigate the nature and role of basic beliefs about the world and oneself that could foster people’s ability to hope in a broader sense, taking special consideration of different cultural backgrounds. According to the definition outlined in the introductory section, the general perception of hope rests on the belief of the possibility, although not probability, that a certain desired good can be attained and the trust in the availability of personal or external resources to overcome difficulties and setbacks (Krafft et al., 2023c). The research question here is: What empowers people to believe that what they hope for can become true? What kind of beliefs sustain people’s general hope? Although basic beliefs are rooted in experiences, they are not necessarily anchored in facts but rather in more generalized assumptions about the world and oneself (Janoff-Bulman, 1992). These worldviews are shaped by cultural norms and values and affect how people think, feel, and act, how they look toward the future, and what kind of wishes, goals, and hopes they consider worthwhile to pursue (Nilsson, 2013, 2014). People in different cultures and contexts may sustain their hope through different basic beliefs. This means that hope could be considered a universal, complex, multifaceted, and at the same time cultural and individual phenomenon (Averill et al., 1990).

The research goal was to investigate whether the culturally shaped basic beliefs of people in two different Swiss regions could have a distinct connection with the levels of perceived hope in their population. The first study aimed to evaluate the role of primal beliefs, as conceptualized and operationalized by Clifton and Yaden (2021), supplemented by the assessment about one’s individual capacity to achieve goals (agency) and overcome difficulties (pathways) as defined by Snyder (2002). In the second study, the role of basic assumptions with regard to the world and oneself as defined and operationalized by Janoff-Bulman (1989, 1992) was assessed.

The first finding was that the general experience of hope as measured by the PHS seems to be conceptualized in a similar way across the French- and the Italian-speaking Swiss population. The French and Italian PHS are reliable instruments to measure perceived hope in a broader sense. Consequently, we could compare the levels of perceived hope and could analyze the correlates and predictors of hope in both samples.

In general terms, one fundamental result is that primal basic beliefs are more strongly related to the broader perception of general hope (PHS) than with the willpower and personal capacity directed to achieving one’s own goals (ADHS). Certainly, the broader perception of hope is significantly related to the self-assessed willpower and ability to achieve one’s own personal goals. However, the belief in the goodness of the world adds significantly to the statistical explanation of the level of perceived hope (PHS). The results in Study 2 confirmed that the more people believe in a benevolent, just, and controllable world and the more they believe in external instances (such as luck) and less in pure chance and unpredictability, the more hopeful they are.

The findings support the assumption that basic beliefs have a significant effect on the level of perceived hope, however with diverse magnitudes in different groups. Two distinct regions in Switzerland with the same economic and political environment but with different languages and cultural backgrounds exhibited significant differences in levels of hope and some basic beliefs. In both studies, the Italian population displayed significantly higher levels of hope, both as measured by the PHS, as well as the ADHS. Since the two population groups are very similar in terms of socio-economic conditions, it could be assumed that the differences in the level of hope might have cultural roots.

The Italian participants displayed higher levels of perceived hope, considering the world to be more just and controllable and themselves as having been luckier in life. Not only do they perceive the future more hopefully, but also envision the world to be more interesting, abundant, improvable, and at the same time less mechanistic or distant. Compared to the French group, the Italian-speaking participants believed more in an enticing and less mechanistic world (Study 1) and were considered to have been luckier in life (Study 2). Observing the predictive power on perceived hope, the French sample revealed a stronger effect of personal disposition to achieve goals than the Italian group. This means that the French-speaking population’s perception of hope is related to a greater extent to the individual’s agency and capacity to achieve personal goals. Instead, in the Italian group the effect size of the primal belief *Good* was considerably stronger than in the French sample. For the Italian-speaking population, the belief in the world as safe, cooperative, and stable has a stronger connection to the general perception of hope than for the French population. These could be possible explanations for why the Italian population reported higher levels of perceived hope.

These findings could suggest that, taking as an example the French-speaking population, the individualistic, cognitive, and internal locus of control could be predominant for the perception of hope as maintained by Snyder (2002), but that this self-centered attitude is sometimes not sufficient to foster higher levels of hope. Without diminishing the importance of self-confidence in terms of agency and pathways, hope, in a broader sense, is much more than just the belief in the individual capacity to achieve one’s own goals. Strong beliefs in the benevolence, justice, abundance, beauty, and livelihood of the world, as well as a sense of luck and self-worth seem to be additional features or sources of hope. Particularly the awareness of having been a lucky person demonstrates that not everything is in our hands and that sometimes we can also trust life (or a Higher Power) in the realization of one’s own hopes.

Several researchers in psychology have already integrated personal and cultural beliefs in the study of hope (Averill et al., 1990; Averill and Sundararajan, 2005; Scioli and Biller, 2009; Tennen et al., 2002). The current studies back the assumption that hope, in a broader sense, is connected to beliefs people might have regarding themselves and the world. The concept of hope presented in this contribution has the advantage to incorporate at a general level of abstraction the notion of belief, and at the same time to address the individual and cultural roots and elements of hope. As in previous research (Krafft et al., 2023b), this study evaluates whether levels of hope vary across cultural groups and examines which basic beliefs could be identified as possible determinants of hope. The findings endorse the idea that how people might hope must be understood in the context of the culture in which the phenomenon is embedded. The question is, what empowers people in certain cultures to believe and hope? What kind of beliefs sustain people’s general hope? Further research could evaluate to what extent certain beliefs could be related to hope in other countries and societies.

The current findings also hold relevant implications for therapists, counselors and practitioners in general. In order to foster hope with regard to one’s own future as well as to the general future of society, practitioners must focus not only on the views of people about their own capabilities, but pay attention to the relationship between the individual and the perception of his/her immediate and broader environment. Interventions directed to improve the mental health and wellbeing of the population might acknowledge the basic and sometimes implicit beliefs people sustain about themselves and the larger environment and their relationship to hope.

## 5 Limitations

The studies in this paper have a number of limitations, which are necessary to address. The cross-sectional design of the research does not allow any conclusions about causalities. Although the demographic structures of the samples are largely heterogeneous, they are not representative of the Swiss population. The participants have been recruited via online platforms, which for some people makes participation more difficult, e.g., for the elderly. Therefore, in order to avoid overgeneralizations about cultural differences, the results of the present studies do not allow to extrapolate cultural norms of the French and Italian speaking populations in Switzerland. Furthermore, it was not the aim of the present studies to perform an ethnographic analysis of cultural differences (e.g., in terms of values) of these two population groups. A limitation already mentioned is the unequal sample sizes, the dissimilar demographic structures, and the lack of representativeness across samples. A further limitation is the internal consistency of some subscales, which was barely acceptable. To avoid potential biases and measurement errors we mainly focused the interpretation on those variables with good internal consistency.

## 6 Conclusion

The findings in this paper support the notion that cultural norms and basic beliefs have an important effect on perceived hope and that people in different cultural contexts sustain hope in different ways. At the same time, there are certain universal elements that foster hope, such as belief in the good and self-confidence (as measured by the ADHS). With regard to psychological theories of hope, these findings imply that it would be misleading to reduce the experience of perceived hope only to individualistic goal-oriented dimensions and to ignore other sources of hope. The concept of hope supported in this paper has the advantage of incorporating, at a general level of abstraction, many dimensions, and at the same time, addressing the individual and cultural roots of hope. Further research could determine if similar patterns emerge in other countries and which cultural characteristics appear in those societies. In addition, much more research is needed to fully understand specific cultural norms, practices, and beliefs in relation to hope.

## Data Availability

The raw data supporting the conclusions of this article will be made available by the authors on request, without undue reservation.
